# White matter microstructure and longitudinal relaxation time anisotropy in human brain at 3 and 7 T

**DOI:** 10.1002/nbm.4815

**Published:** 2022-09-12

**Authors:** Risto A. Kauppinen, Jeromy Thotland, Pramod K. Pisharady, Christophe Lenglet, Michael Garwood

**Affiliations:** 1 Department of Electric and Electronic Engineering, University of Bristol, Bristol, UK; 2 Center for Magnetic Resonance Research, University of Minnesota, Minneapolis, Minnesota, USA

**Keywords:** microstructure, relaxation anisotropy, T1 relaxation, white matter

## Abstract

A high degree of structural order by white matter (WM) fibre tracts creates a physicochemical environment where water relaxations are rendered anisotropic. Recently, angularly dependent longitudinal relaxation has been reported in human WM. We have characterised interrelationships between T1 relaxation and diffusion MRI microstructural indices at 3 and 7 T. Eleven volunteers consented to participate in the study. Multishell diffusion MR images were acquired with b-values of 0/1500/3000 and 0/1000/2000 s/mm^2^ at 1.5 and 1.05 mm^3^ isotropic resolutions at 3 and 7 T, respectively. DTIFIT was used to compute DTI indices; the fibre-to-field angle (*θ*_FB_) maps were obtained using the principal eigenvector images. The orientations and volume fractions of multiple fibre populations were estimated using BedpostX in FSL, and the orientation dispersion index (ODI) was estimated using the NODDI protocol. MP2RAGE was used to acquire images for T1 maps at 1.0 and 0.9 mm^3^ isotropic resolutions at 3 and 7 T, respectively. At 3 T, T1 as a function of *θ*_FB_ in WM with high fractional anisotropy and one-fibre orientation volume fraction or low ODI shows a broad peak centred at 50°, but a flat baseline at 0° and 90°. The broad peak amounted up to 7% of the mean T1. At 7 T, the broad peak appeared at 40° and T1 in fibres running parallel to B0 was longer by up to 75 ms (8.3% of the mean T1) than in those perpendicular to the field. The peak at 40° was approximately 5% of mean T1 (i.e., proportionally smaller than that at 54° at 3 T). The data demonstrate T1 anisotropy in WM with high microstructural order at both fields. The angular patterns are indicative of the B0-dependency of T1 anisotropy. Thus myelinated WM fibres influence T1 contrast both by acting as a T1 contrast agent and rendering T1 dependent on fibre orientation with B0.

## Graphical Abstract

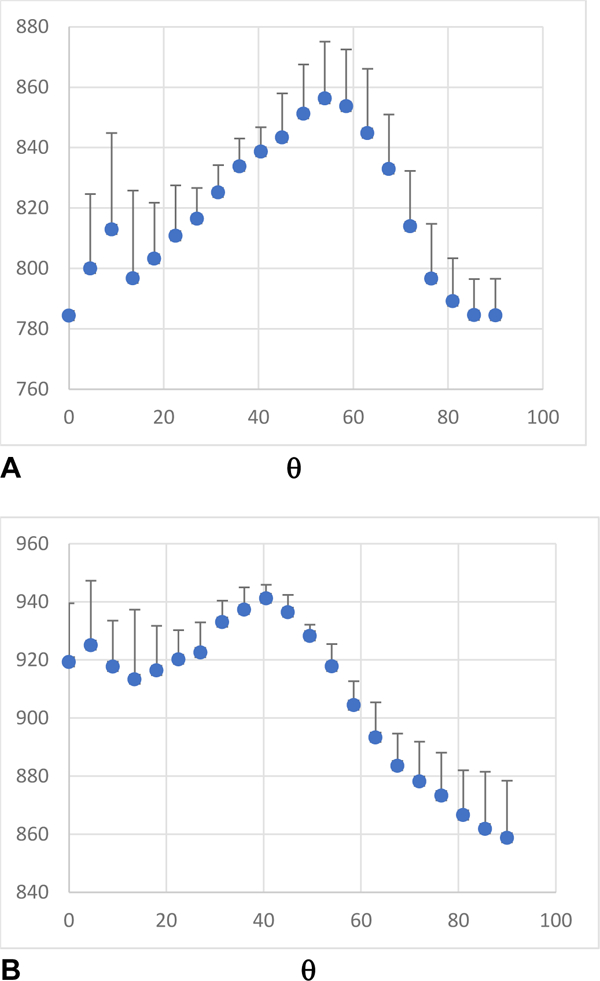

White matter (WM) with high degree of structural anisotropy, with single fibre orientation, shows angular dependency of T1 with B0.

At 3T a broad peak in T1 centred at 54° and flat baseline across 0° and 90° are seen in WM with orientation dispersion index (ODI) of 0.05 (Figure A, T1 as a function of fibre-to-filed-angle (θ)).

At 7T the broad T1 peak is centred at 40°, when T1 in fibres running parallel to field is longer than in those perpendicular to B0 (Figure B, T1 vs θ for WM with ODI=0.05).

These data show B0-dependency of angular T1 patterns in WM and link axonal fibre orientation with B0 to T1 MRI contrast.

## 1 | INTRODUCTION

A striking contrast between grey matter (GM) and white matter (WM) in T1-weighted MRI images is widely exploited in neuroimaging for volumetry, cortical thickness and shape analyses, as well as in clinical neuroimaging. At the clinical field strength of 3 T, T1 relaxation time as measured by inversion recovery-based methods in GM varies from 1100 to 1600 ms, and in WM from 650 to 900 ms.^1,2^ The T1 gradient between GM and WM, the underpinning of T1 contrast, is due to several factors, such as water content, myelination and the extracellular-to-intracellular volume ratio.^3–5^ While greater water and lower myelin contents in GM may explain its longer T1 compared with WM T1, thorough understanding of the physical underpinnings of WM T1 is lacking.^5,6^ Nevertheless, it is firmly established that T1 in WM is inversely proportional to myelin content,^7,8^ and that water proton exchange between free and macromolecular sites is a major determinant of T1.^5,9^ In addition, compartmentation of water within and between axons creates physicochemical environments where water mobility and interactions with nonaqueous species vary greatly. For instance, myelin-associated water in central nervous system axons has been shown to behave in a liquid crystal-like fashion.^10^

T1 relaxation in WM has been commonly modelled using a ‘myelinated lattice’ (i.e., axon fibres) and bulk water undergoing magnetisation transfer (MT), both via a through-space dipolar interaction and magnetisation exchange mediated by protons, the so-called ‘binary spin bath model’.^11,12^ In the binary spin bath model, two pools of protons are involved, one semisolid (nonaqueous) and one aqueous, with characteristic relaxation rates for protons and exchange rates. A further development of the binary spin bath model is a four-pool model^6^ where the nonaqueous protons are considered both in myelin and nonmyelin compartments, and aqueous protons both in the myelin compartment and intracellular/extracellular spaces. Experimentally, it has been shown that B0-dependent short (~100 ms or so) and B0-independent long (several hundred milliseconds to seconds) T1 components exist in WM,^13^ whereby the short T1 water is tentatively assigned to nonaqueous species and/or myelin-associated water and the long T1 is linked to aqueous pools, including intracellular/extracellular water pools.^6,14^

In addition to the mechanisms described above, recent evidence points to multiple effects of WM microstructure on MRI T1 relaxation. In the corpus callosum (CC), T1 has been shown to be longer in somato-motor areas where axon density is lower and the proportion of large (up to 9 μm in diameter) axons^15^ is higher than in other areas (e.g., genu and splenium) where tightly packed axon fibres are typically less than 1 μm.^16,17^ The high degree of structural order of those WM fibre tracts creates physicochemical environments where water relaxations are rendered anisotropic with respect to B0.^18–20^ Diamagnetic susceptibility gradients across WM fibres and their immediate surroundings impact transverse relaxation.^21^ Specifically, susceptibility anisotropy in WM results in accelerated transverse relaxation in fibres perpendicular to B0.^19,21^ Recently, angularly dependent longitudinal relaxation has been observed in human WM.^22,23^ In a study using both a variable flip angle (VFA) method and an inversion recovery sequence with a turbo-spin echo readout for T1 mapping, Schyboll et al. found T1 values at 3 T in fibres running parallel to B0 to be approximately 2%–3% longer than those in fibres running perpendicular to B0.^22^ At variance with this angular pattern, longer T1 around the fibre-to-field angle (*θ*_*FB*_) of 50° by 4% in WM with high fractional anisotropy (FA) was observed in MP2RAGE,^23^ but no difference between 0° and 90° fibre orientations. The magnitude of the angular dependence of T1 values reduces in the brain of older individuals,^22,23^ indicating that anisotropic T1 data may be a useful additional neuroimaging marker to diffusion MRI (dMRI) indices to WM microstructure integrity.

Here, we have further characterised T1 anisotropy, in relation to dMRI-derived WM microstructural indices, and have studied the plausible B0 dependency of T1 anisotropy. Multishell dMRI was acquired both at 3 and 7 T in the same participants to estimate WM microstructural indices beyond FA including the orientation dispersion index (ODI) and volume fractions of primary, secondary and tertiary fibre bundles, together with T1 maps measured using the MP2RAGE sequence in young adult volunteers.

## 2 | EXPERIMENTAL

### 2.1 | Human subjects

The study received ethical approval from the University of Minnesota Institutional Review Board. Eleven volunteers with no history of neurological conditions (mean age 27.5 years, range 23–35 years; five females) consented to participate in the study. All 11 volunteers were scanned at 3 T. Six of them (mean age 27 years, two females, within 4 months after 3-T scans) were also scanned at 7 T.

### 2.2 | MRI

A Siemens MAGNETOM Prisma 3-T system with a 32-channel head coil and a Siemens MAGNETOM 7-T scanner with a Nova Medical 1-transmit/32-receive head coil were used. At 3 T, dMR images were acquired using the Human Connectome Project (HCP) Lifespan Protocol^24^ with the parameters given in Table 1. A B0 field map was acquired using a spin echo EPI sequence with TR = 8000 ms, TE = 66 ms and 2 mm^3^ isotropic resolution. A B1 map was also acquired at a resolution of 4 × 4 × 8 mm^3^ using the manufacturer’s routine. At 7T, dMR images were acquired using the HCP Young Adult Protocol^25^ with the parameters shown in Table 1.

An MP2RAGE sequence was used to acquire images for T1 mapping both at 3 and 7 T. At 3 T, the acquisition parameters were as follows: TR = 2000 ms, TE = 1.68 ms, adiabatic inversion pulse, hard rectangular 4° read pulse, six TIs of 200, 300, 600, 900, 1200 and 1500 ms (where TI is defined as the time from the beginning of the inversion pulse to the phase encode centre line), 1.0 mm^3^ isotropic resolution, GeneRalized Autocalibrating Partial Parallel Acquisition (GRAPPA) 3, phase partial Fourier 6/8, slice partial Fourier 6/8, linear phase encoding, in three scan blocks with a scan time of 2 min 35 s each. At 7 T, the acquisition parameters were as follows: TR = 6500 ms, TE = 1.49 ms, adiabatic inversion pulse, hard rectangular 4° read pulse, six TIs of 300, 600, 1000, 1500, 2000 and 3000 ms, 0.9 mm^3^ isotropic resolution, linear phase encoding, GRAPPA 3, phase partial Fourier 6/8 and slice partial Fourier 6/8, in three scan blocks with a scan time of 9 min 14 s each. Anatomical T1-weighted MPRAGE images at both fields (acquired at 0.8 and 1.0 mm^3^ isotropic resolutions at 3 and 7 T, respectively) were used to segment GM, WM and cerebrospinal fluid spaces.

### 2.3 | Image processing

dMRI scans were corrected for distortions due to eddy currents, susceptibility-induced off-resonance artifacts and subject motion using TOPUP and EDDY in FSL.^26,27^ A DTI model was subsequently fitted to the corrected data using DTIFIT in FSL,^28^ to compute the DTI indices [FA, mean diffusivity (MD), V_1_, V_2_, V_3_] using b = 0 and b = 1500 s/mm^2^ images at 3 T and b = 0 and b = 1000 s/mm^2^ images at 7 T. The option of fitting the tensor to the weighted least squares was used in DTIFIT. The general consensus is that the optimal b-value lies within 700 and 1500 s/mm^2^, with 1000 s/mm^2^ being the most commonly used value.^29^ Fibre-to-field angle maps were computed using the principal eigenvector V_1_ images as described elsewhere.^23^ The orientations and volume fractions of multiple fibre populations (first fibre = F1, second fibre = F2 and third fibre volume fraction = F3) were estimated using BedpostX in FSL.^30^ The neurite orientation and density imaging (NODDI) pipeline in Matlab^31^ was employed to create ODI maps. The NODDI approach recovers neurite orientation and density using a single Watson distribution. However, the NODDI approach accounts poorly for crossing fibre configurations.^32^ Nevertheless, we chose to use the NODDI technique because the resulting ODI maps can be directly used in conjunction with DTI-derived fibre-to-field angle images. Furthermore, it was concluded recently that crossing fibre bias to fibre-to-field angle definitions concerns WM where FA is less than 0.7.^33^ Our quantitative T1 orientation data are derived chiefly from WM with FA more than 0.7.

The signal-to-noise ratio (SNR) was computed for diffusion images using the procedure described in^34^ as implemented in DIPY.^35^ A mask encompassing the midsagittal portion of the entire length of the CC in the anterior-posterior direction was used to measure SNR in images (Figure S2). It should be noted that the worst case scenario of SNR applies to the X-gradient direction, because the CC is positioned close to the X direction, thereby receiving the most attenuated signal. It is evident from Figure S2 that even in the worst case scenario the SNRs in b = 3000 s/mm^2^ for 3-T and b = 2000 s/mm^2^ for 7-T images were above the noise floor bias.^36^

T1 and S0 (a proxy for M0 obtained from zero TI of the MP2RAGE data fits) maps were computed as previously described^23^ and registered to the FA images using FLIRT in FSL.^37^ 1D and 2D plots of T1 and S0 as a function of *θ*_*FB*_ and one of either the DTI, BedpostX or NODDI indices were computed in Matlab as previously described.^23^

## 3 | RESULTS

**FIGURE 1:**
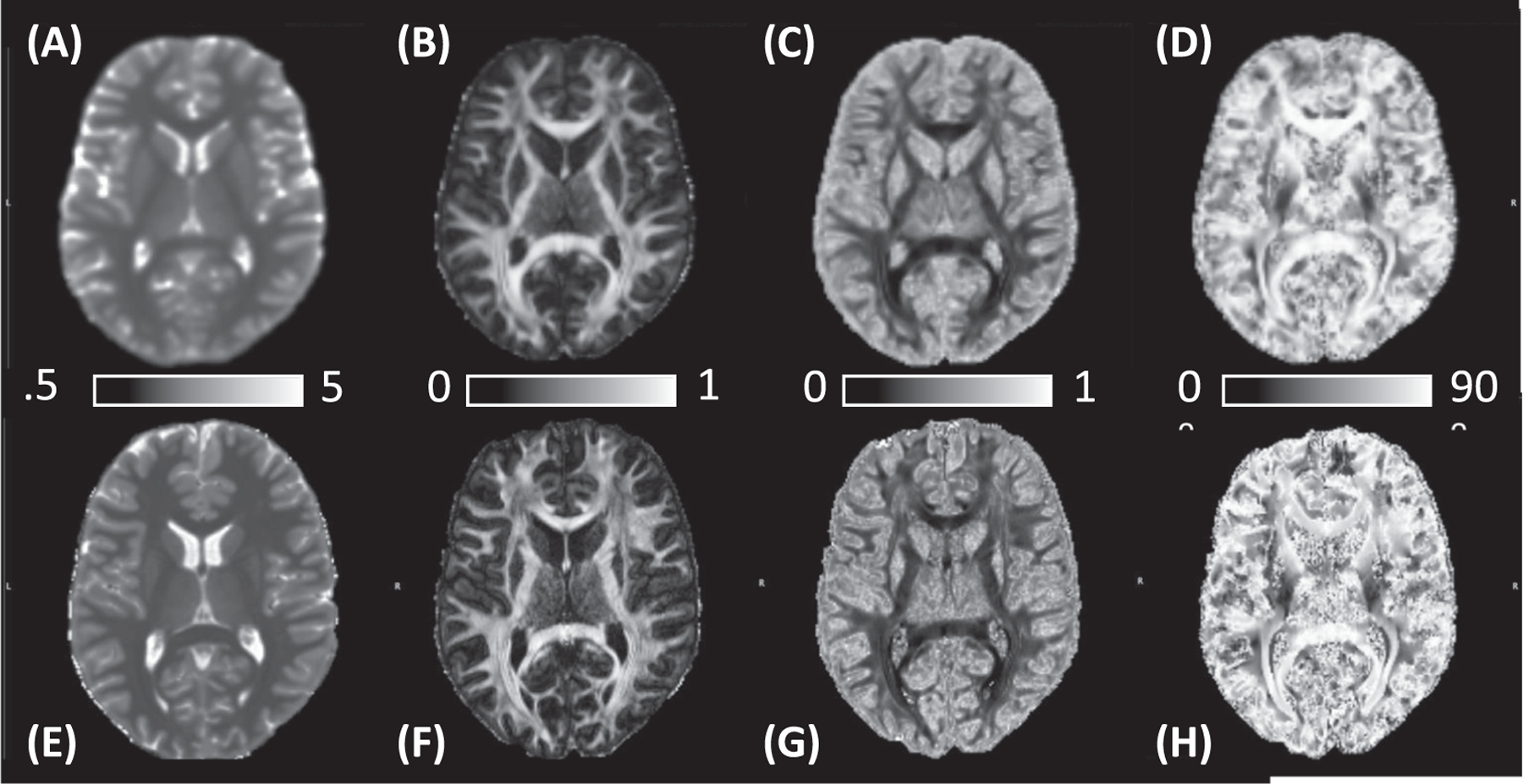
Typical axial parametric maps from a 26-year-old male volunteer scanned both at (A–D) 3 T and (E and F) 7 T. (A) and (E) are T1 (scale bar in seconds), (B) and (F) Fractional anisotropy (FA) (scale bar from minimum to maximum), (C) and (G) Orientation dispersion index (ODI) (scale bar from minimum to maximum) and (D) and (H) Fibre-to-field maps (scale bar in degrees)

Typical T1 maps, FA, ODI and *θ*_*FB*_ images at 3 and 7 T are shown (Figure 1). The mean T1 in WM with FA ranging from 0.45 to 0.9 (the range of focus in the paper) was 829.3 ± 25.8 ms at 3 T and 917.8 ± 22.2 ms at 7 T.

**FIGURE 2:**
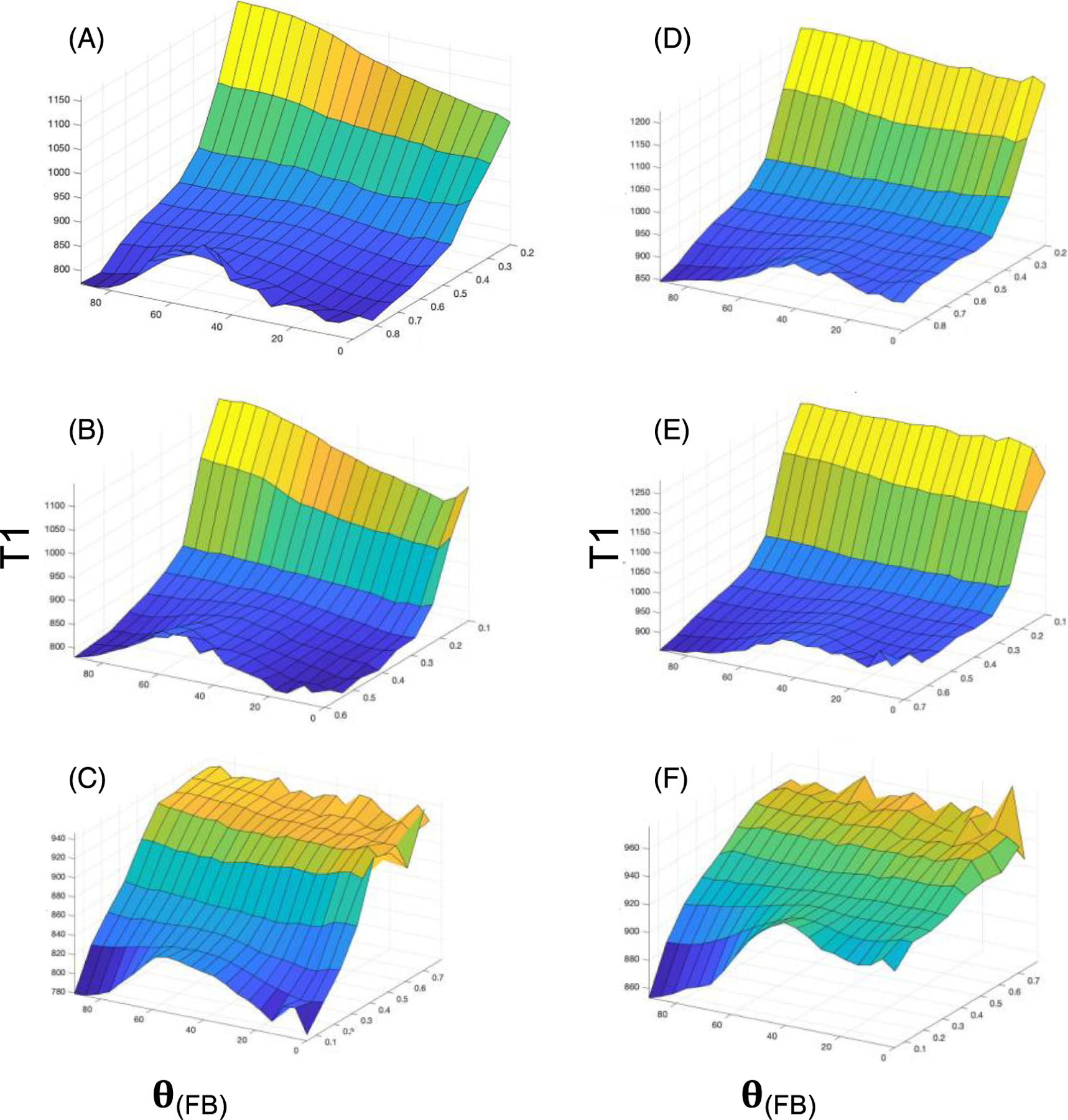
2D plots for T1 at (A–C) 3 T and (D–F) 7 T as a function of fibre-to-field angle (**θ**) and given microstructural MRI index as indicated. (A) Data from fractional anisotropy (FA), (B) One-fibre orientation volume fraction (F1) and (C) Orientation dispersion index (ODI) acquired at 3 T. Data from 3-T MRI are representative for all 11 volunteers. (D) Data from FA, (E) F1 and (F) ODI acquired at 7 T. Data from 7-T MRI are representative for all six volunteers. T1 is given in ms; **θ**_**(FB)**_ = fibre-to-field angle in degrees. Note than in (C) and (F) the transverse axis is in ascending order

**FIGURE 3:**
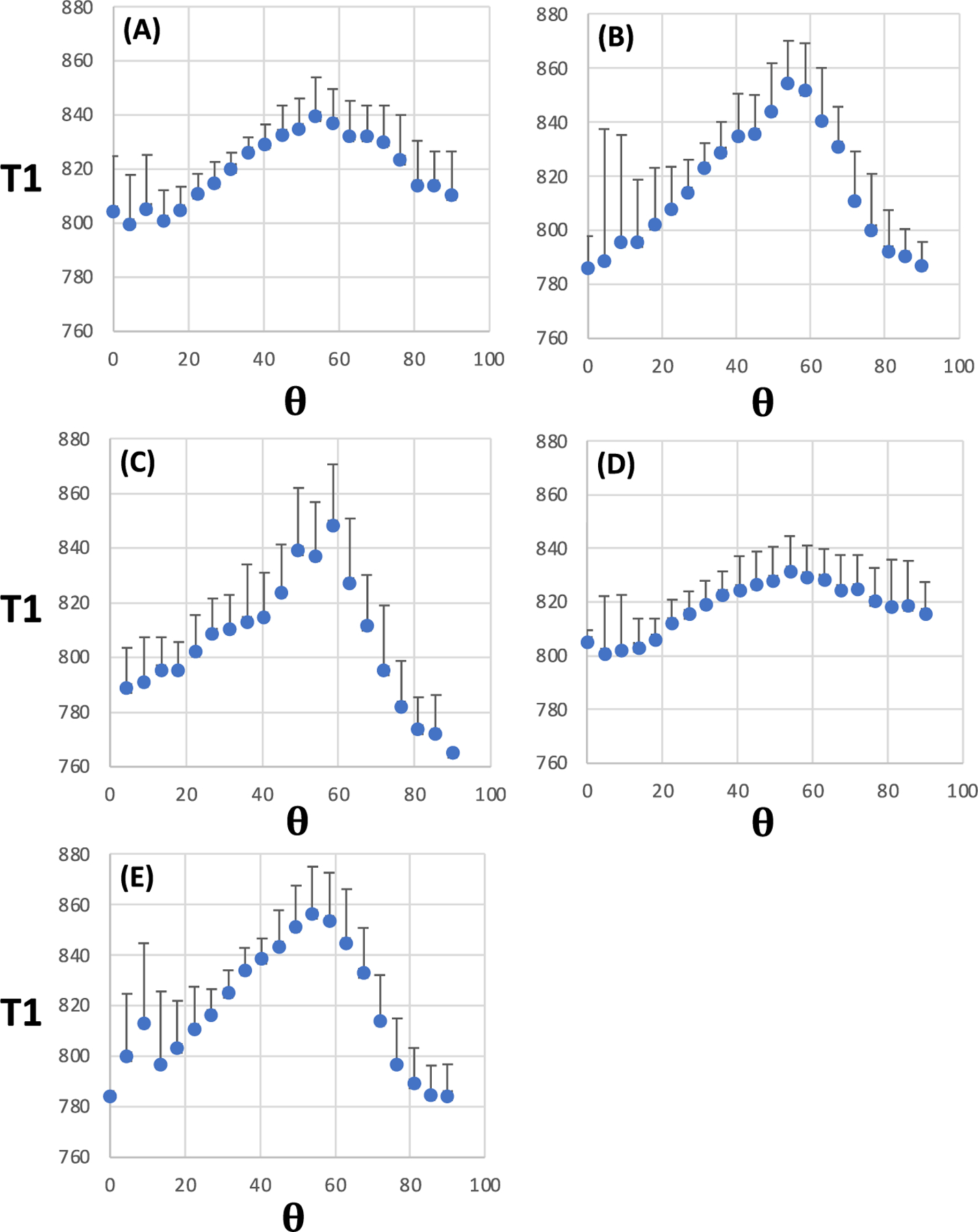
1D plots for T1 as a function of fibre-to-field angle in white matter selected for a given microstructural MRI index. (A) Fractional anisotropy (FA) (median FA = 0.725); (B) One-fibre orientation volume fraction (F1) (median F1 = 0.52); (C) Two-fibre orientation volume fraction (F2) (median F2 = 0.31); (D) Three-fibre orientation volume fraction (F3) (median F3 = 0.24) and (E) Orientation dispersion index (ODI) (median ODI = 0.05). Data are from 3-T MRI and are shown as mean ± SD (n = 11). T1 is given in ms; **θ** = fibre-to-field angle in degrees

2D plots for WM T1 at 3 T are shown as a function of *θ*_*FB*_ and FA (Figure 2A), F1 (Figure 2B) and ODI (Figure 2C). A broad peak in T1 centred at *θ*_*FB*_ of ~50° is evident in the panels for WM with a high degree of structural anisotropy (i.e., at high FA and F1) or a low fibre dispersion (i.e., low ODI). T1 profiles at the intermediate and low ends of FA and F1 as well as high and intermediate ends of ODI were remarkably flat across the angular ranges. Further details on T1 values for WM voxels with high FA, F1, F2 and F3, as well as low ODI, confirm the broad peak in T1 at an *θ*_*FB*_ of 54° (Figure 3). The 1D plots revealed no difference in T1 values for *θ*_*FB*_ of 0° and 90° (Figure 3).

**FIGURE 4:**
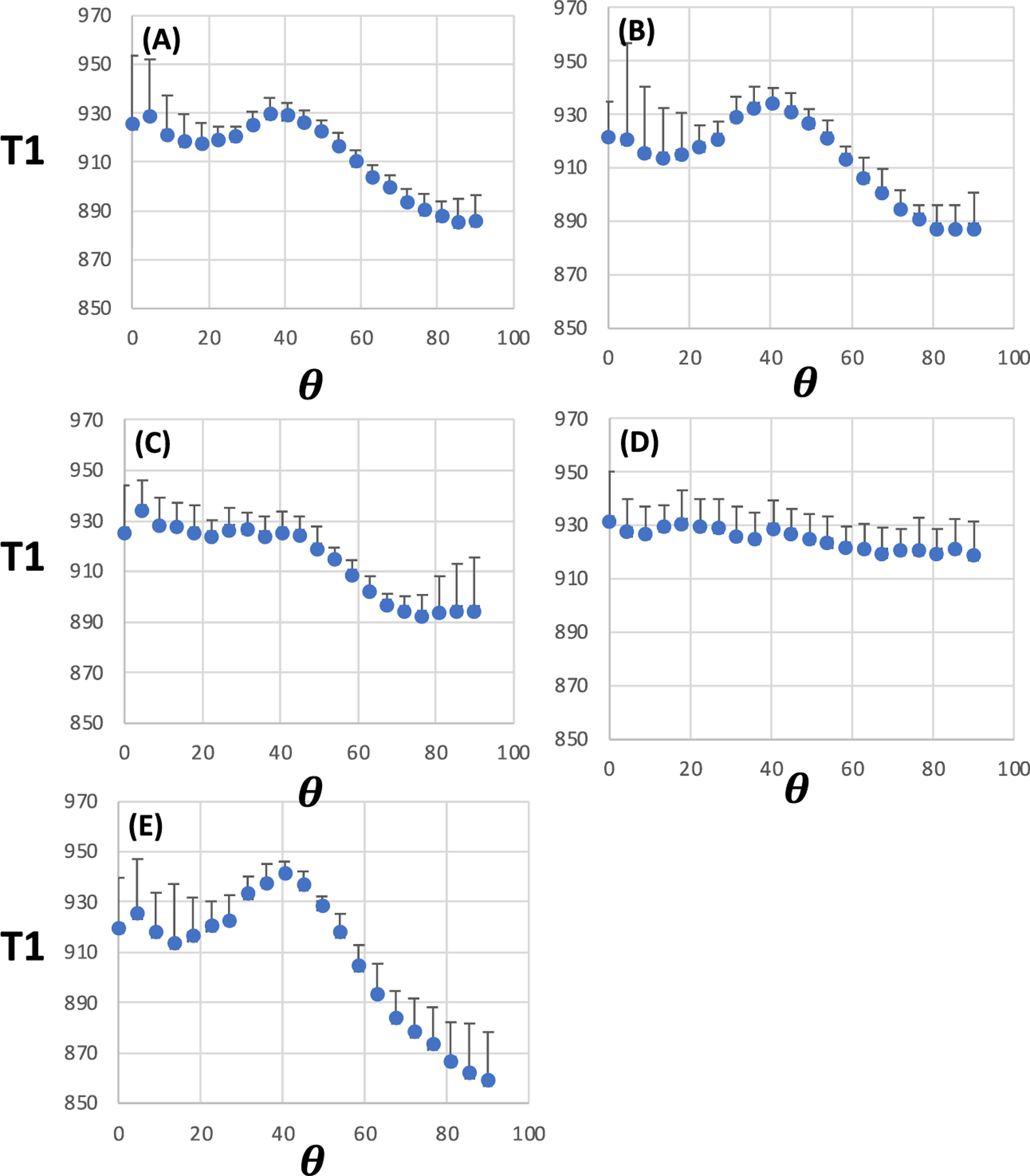
1D plots for T1 as a function of fibre-to-field angle in white matter selected for a given microstructural MRI index. (A) Fractional anisotropy (FA) (median FA = 0.725); (B) One-fibre orientation volume fraction (F1) (median F1 = 0.55); (C) Two-fibre orientation volume fraction (F2) (median F2 = 0.33); (D) Three-fibre orientation volume fraction (F3) (median F3 = 0.21) and (E) Orientation dispersion index (ODI) (median ODI = 0.05). Data are from 7-T MRI and are shown as mean ± SD (n = 6). T1 is given in ms; *θ* = fibre-to-field angle in degrees

2D plots for WM T1 at 7 T as a function of *θ*_*FB*_ and microstructural dMRI indices are shown (Figure 2D-F). 2D plots for FA (Figure 2D), F1 (Figure 2E) and ODI (Figure 2F) as an index of structural order demonstrated angularly dependent T1 in highly anisotropic WM. It was observed that the 2D plots show two notable differences in the angular dependency of T1 compared with 3-T data: first, the broad T1 peak appeared at around 40° of *θ*_*FB*_ instead of 54°, and second, T1 values at the *θ*_*FB*_ = 0° end were longer than at the 90° end. 1D plots of T1 as a function of *θ*_*FB*_ (Figure 4) for each WM microstructural index clearly demonstrate the broad peak at 40° and a positive gradient in T1 between 0° and 90° in FA (Figure 4A), F1 (Figure 4B) and ODI (Figure 4E).

To examine whether the differential angular dependency of T1 at 7 T could be a result of the higher spatial resolution used at this field, MRI data from six volunteers scanned at both fields were examined independently. T1 data at 3 and 7 T for WM with high FA (Figure S1) show the peaks at 54° and 40°, respectively. When 7-T T1 maps were downsampled to the spatial resolution used at 3 T and registered to the 3-T FA maps from the same subjects, identical angular plots were obtained as those observed with the original 7-T data (Figure S1). These data argue that the observed differences in T1 angular patterns at 3 and 7 T are likely inherent to the T1 relaxation process at each field.

Table 2 provides quantitative data for T1 and microstructural indices. The mean T1 value at 3 T varied between 804.7 and 817.9 ms in the WM analysed for various MRI microstructural indices. T1 and FA values in WM tissue shown for ODI, F1 and F2 (Figure 3) were in the same range as in WM selected by FA only (Table 2). The average T1 peak at 54° in the 1D ODI plot was 59.4 ms, as opposed to that in FA, which was 38.7 ms. However, in WM for F3 analyses, FA was lower than in the four other datasets. No consistent T1 differences between 0° and 90° fibre angles were seen for any MRI microstructural indices at 3 T (Table 2). The mean T1 at 7 T ranged from 905.0 to 924.9 ms in the analysed WM (Table 2). The amplitude of T1 peaks (at an *θ*_*FB*_ of 40°) was smaller at 7 T than that observed at 54° at 3 T. Given that T1 was longer at 7 T, the proportional T1 lengthening at 40° amounted to 4.1% at 7 T as opposed to 7.3% at 54° at 3 T in ODI data. For FA, the T1 peak at 54° was 4.7% of the mean T1 at 3 T in the voxels analysed; the respective percentage at 7 T for the 40° T1 peak was 2.5%. Table 2 shows that T1s at 7 T were longer by ~50–75 ms at 0 than at 90 in WM selected by FA and ODI.

**FIGURE 5:**
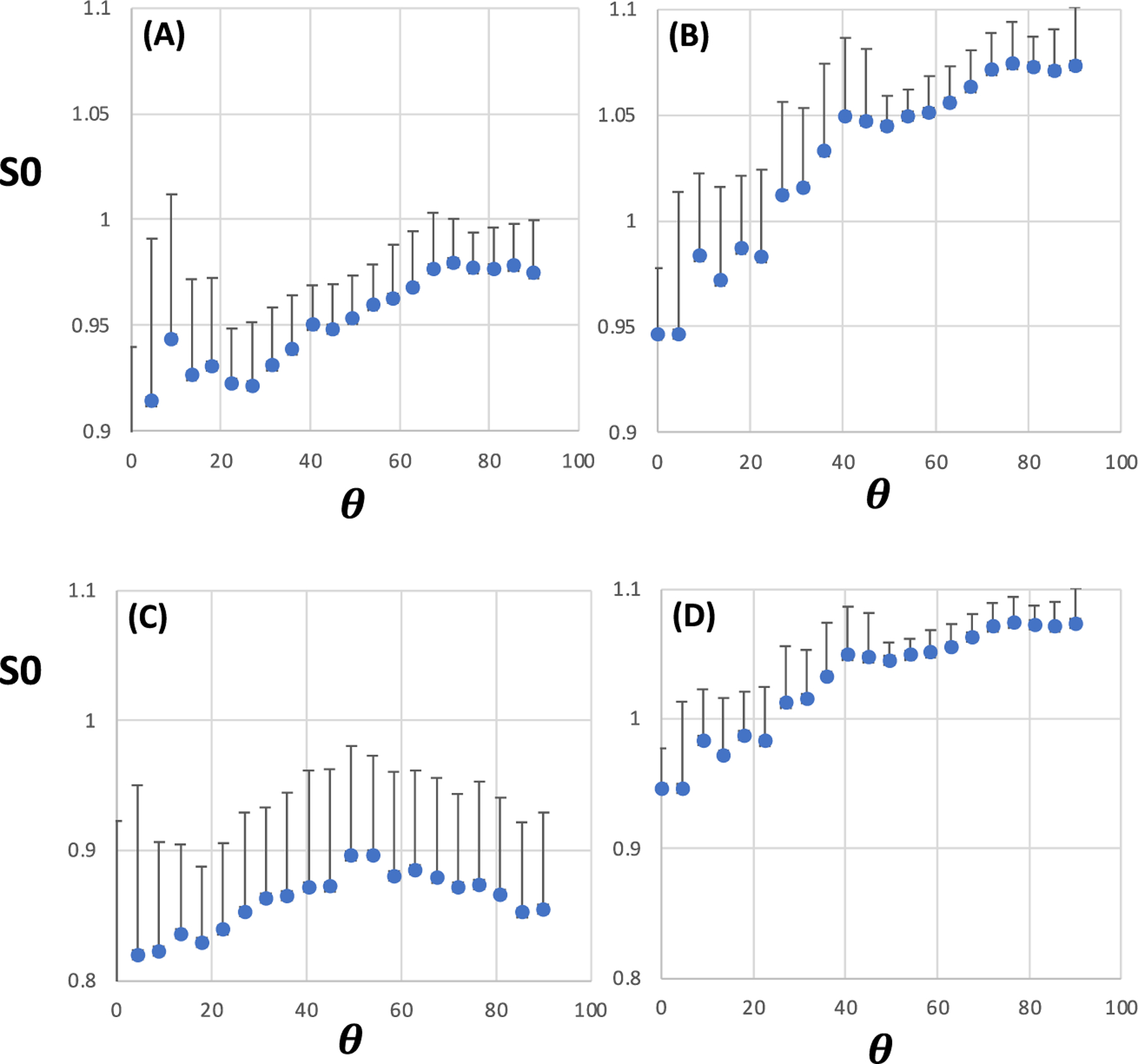
1D plots for normalised S0 as a function of fibre-to-field angle in white matter with median fractional anisotropy (FA) = 0.725 (A and C) and median orientation dispersion index (ODI) = 0.05 (B and D). In (A) and (C), S0 was normalised for baseline using the intensity from voxels with FA = 0.2, and in (C) and (D) from voxels with ODI = 0.7. (A) and (B) are from 3-T MRI (n = 11); (C) and (D) are from 7-T MRI (n = 6); *θ* = fibre-to-field angle in degrees

To evaluate the proton density (PD) as a function of *θ*_*FB*_, the normalised S0s derived from MP2RAGE data fits were used as a proxy for high FA and low ODI WM. The 1D S0 plots for FA and ODI showed no resolved peak at any *θ*_*FB*_ at either field (Figure 5), but a tendency to higher S0 values in the 90° end compared with 0° was evident in the plots at both fields. The S0 plots show qualitative resemblance to those reported by Schyboll et al.,^22^ but a disconnect with the angular patterns of T1 shown in Figures 3 and 4.

**FIGURE 6:**
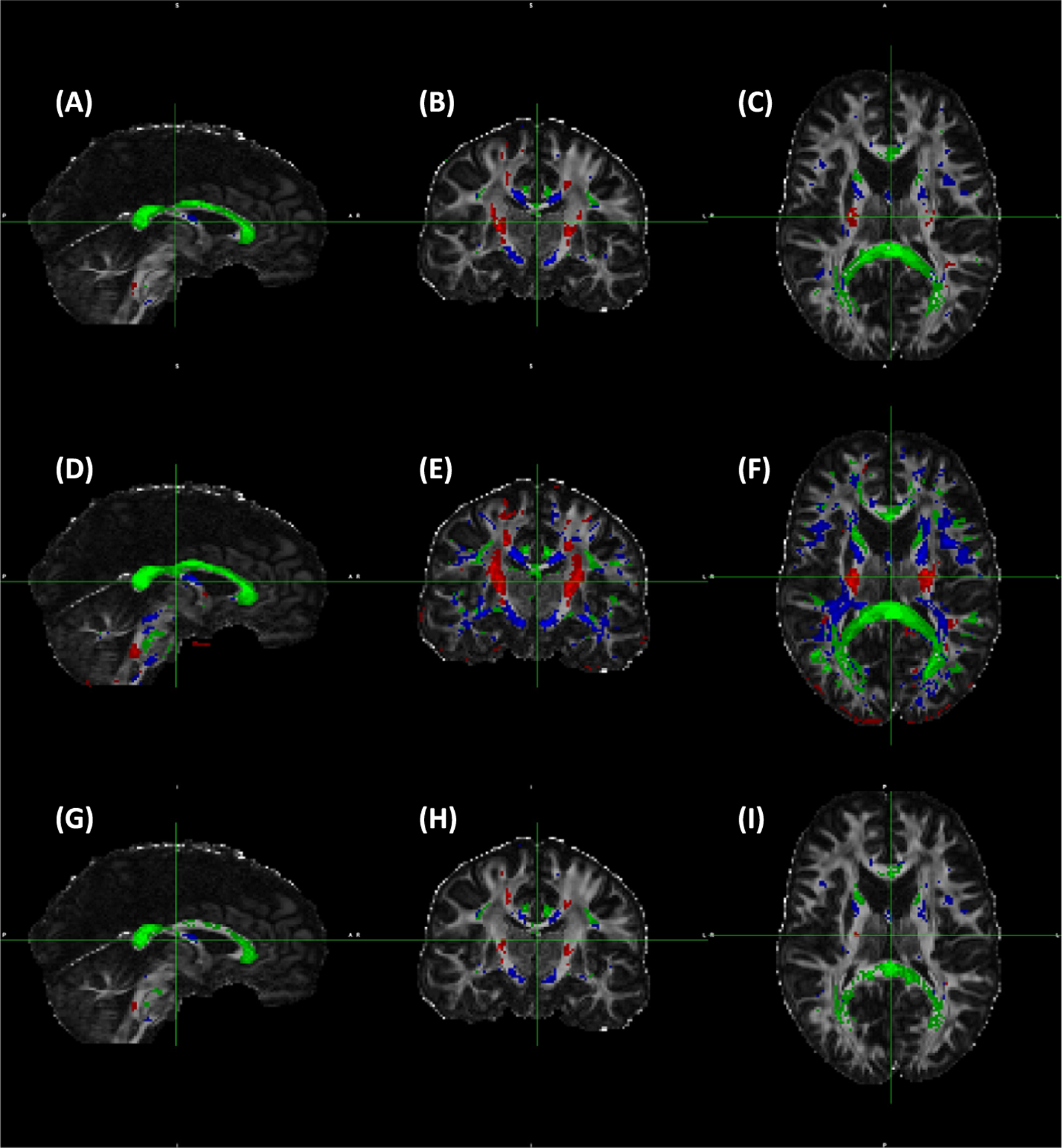
Anatomical distribution of white matter (WM) areas selected according to the fibre-to-field angle. (A–C) Fractional anisotropy (FA) (FA = 0.7–0.9); (D–F) Orientation dispersion index (ODI) (ODI = 0–0.2); and (G–I) One-fibre orientation volume fraction (F1) (F1 = 0.45–0.8). Data are from 3 T and representative to all six volunteers scanned both at 3 and 7 T. Colour coding for each fibre-to-field angle bin is as follows: red: 10° (range 0°–15°), blue 50° (range 45°–63°) and green 90° (range 80°–90°). Volumes of WM (given in mm^3^) are as follows: FA: 10°, 3905 mm^3^; 50°, 19,015 mm^3^; 90°, 17,928 mm^3^. ODI: 10°, 22,345 mm^3^; 50°, 110,845 mm^3^; 90°, 61,867mm^3^. F1: 10°, 1796 mm^3^; 50°, 9619 mm^3^; 90°, 14,608 mm^3^

**FIGURE 7:**
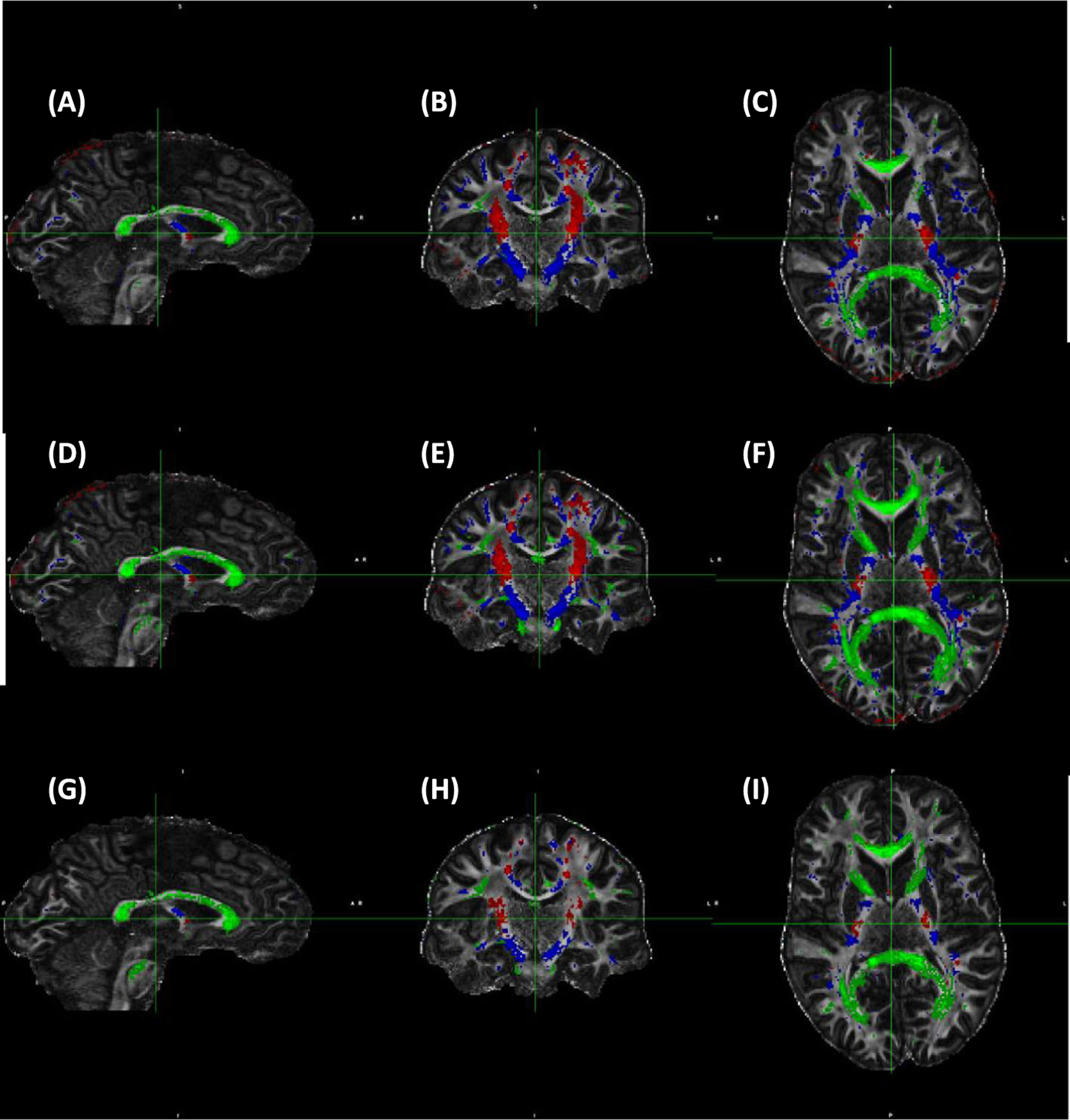
Anatomical distribution of white matter (WM) areas selected according to the fibre-to-field angle. (A–C) Fractional anisotropy (FA) (FA = 0.7–0.9); (D–F) Orientation dispersion index (ODI) (ODI = 0–0.2); and (G–I) One-fibre orientation volume fraction (F1) (F1 = 0.45–0.8). Data are from 7 T and representative to all six volunteers scanned both at 3 and 7 T. Colour coding for each fibre-to-field angle bin is as follows: red: 10° (range 0°–15°), blue 40° (range 35°–50°) and green 90° (range 80°–90°). Volumes of WM (given in mm^3^) are as follows: FA: 10°, 5054 mm^3^; 40°, 69,147 mm^3^; 90°, 17,850 mm^3^. ODI: 10°, 24,244 mm^3^; 40°, 69,147 mm^3^; 90°, 50,906 mm^3^. F1: 10°, 6938 mm^3^; 40°, 24,290 mm^3^; 90°, 29,846 mm^3^

The anatomical distribution of WM voxels binned to three *θ*_*FB*_ of 0–15°, 45–63° and 80–90° for 3 and 7 T for FA, ODI and F1 are shown (Figures 6 and 7). The voxels where fibres run close to parallel to B0 were chiefly located, as expected, in the cortico-spinal tract (CST), and those close to perpendicular to B0 in the CC. Tracts around 50° were scattered throughout the WM (Figure 6), as were those around 40° (Figure 7). These included, for instance, the fornix, lateral aspects of the CC and parts of the anterior limb of the internal capsule. Anatomical coverage of WM contributing to FA, ODI and F1 data showed a high degree of overlap at both fields. It is clear that the WM volumes selected by ODI were larger than those by FA or F1 at both fields; for instance, at 3 T, F1 voxels represent subsets of WM used for ODI (Figure 6D–F vs. G–I). Therefore, the data for angular dependencies presented in Figures 2–5 and Table 2 originated from largely similar WM regions for each *θ*_*FB*_ bin, although for both FA and F1 the subregions were analysed for ODI.

**FIGURE 8:**
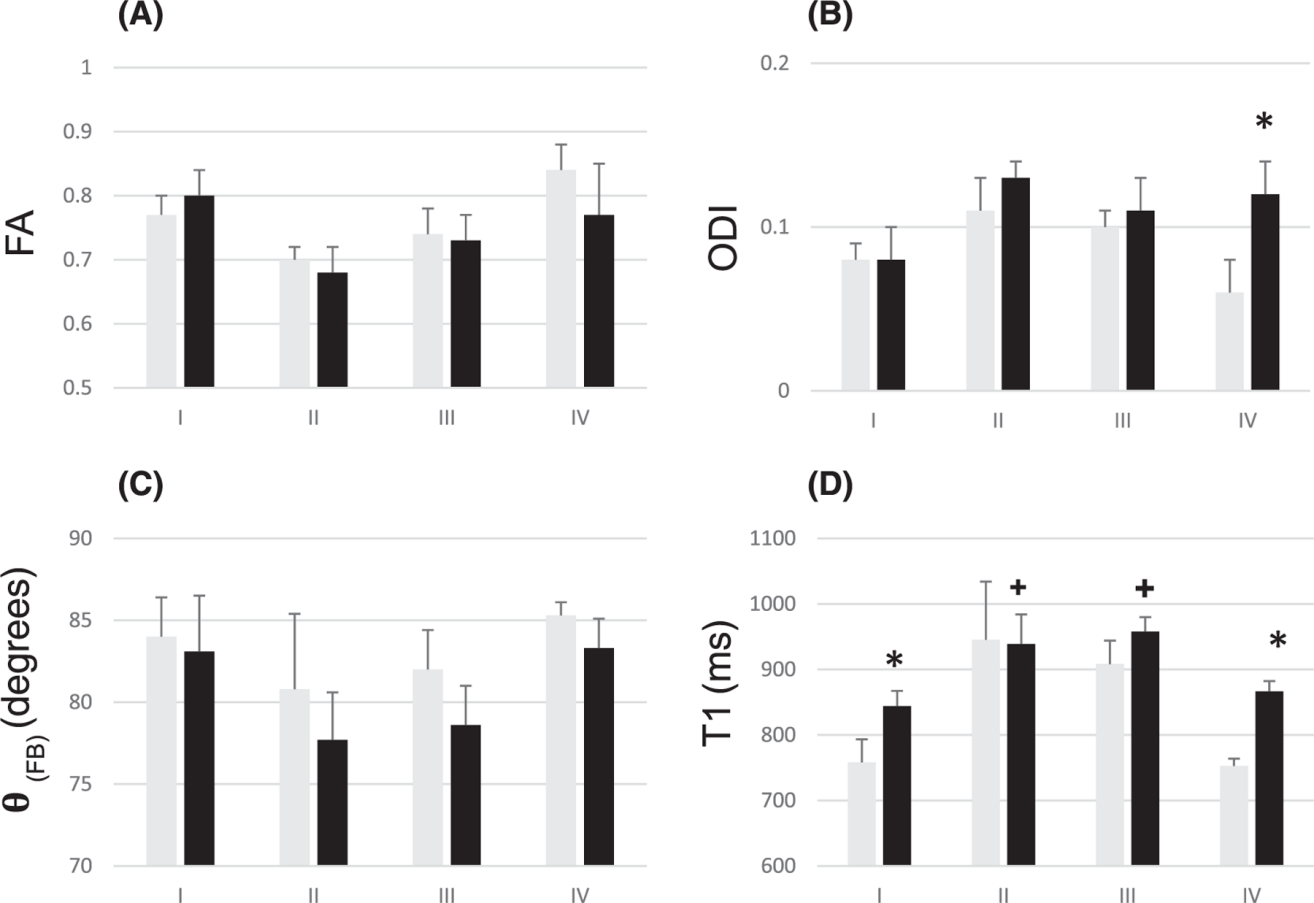
(A–C) Diffusion microstructure index and (D) T1 data for four regions of interest (ROIs) in the corpus callosum (CC) at 3 and 7 T. Rectangular ROIs were placed to the midline (± 2–3 mm on both sides) of the CC in fractional anisotropy (FA) images in genu (I), mid-motor (II), somato-motor (III) and splenium (IV), dimensions 3–5 mm anterior-posterior and 3–5 mm left-right directions. (A) FA, (B) Orientation dispersion index (ODI), (C) Fibre-to-field angle and (D) T1 data from the selected ROIs (I–IV) were determined for six volunteers scanned at both fields. Values are mean ± SD. T1 is given in ms. Student’s *t*-test is used for T1 in ROIs at each field; the Bonferroni-corrected *p* value was set at 0.005. An asterix (*) shows a significant difference between 3 and 7 T; + indicates significance between ROIs

We also analysed T1 values in four different midline sections of the CC, where axon density and diameters are known to vary,^15^ yet fibre orientation is rather unform. Electron microscopy analyses of human CC have revealed that up to 55% of axons in the somato-motor area are larger than 1 μm in diameter (up to 9 μm) in contrast to those in the genu, where 75% of fibres are smaller than 1 μm.^15^ Figure 8 summarises dMRI microstructural indices and T1s in four areas of the CC at both 3 and 7 T. Microstructural indices show high FA and low ODI in all regions of interest (ROIs) and that *θ*_*FB*_ values were typically 80° or higher (i.e., fibres running close to perpendicular to B0). It is noteworthy that ODI in ROI IV at 3 T was lower than that at 7 T. T1s in genu and splenium ROIs were of similar magnitude to each other, both at 3 and 7 T. Significantly longer T1s in ROIs from the midbody and somato-motor areas were seen than in genu at both fields. At 3 T, T1 in the somato-motor ROIs was ~150 ms longer than in genu, while at 7 T the respective T1 difference was ~90 ms. These data show that T1 was longer in areas where axon density is low and the proportion of large diameter fibres is high, rather than in areas where small fibres (<1 μm) dominate, in agreement with the study by Hofer and associates.^16^

## 4 | DISCUSSION

We show interrelationships between microstructural indices derived from dMRI and T1 values in human WM at two magnetic field strengths. The data demonstrate the presence of T1 anisotropy in WM where tissue microstructural indices show high structural anisotropy (i.e., high FA and dominance of a single fibre population or low ODI). T1 anisotropy is observable widely in WM. We show that, at a standard dMRI spatial resolution at 3 T, WM tracts selected by both ODI and F1 (i.e., FA values ~0.7) have a larger T1 peak at *θ*_*FB*_ of 54° than those with the highest FA (~0.7). Our current data also show that T1 anisotropy is B0 dependent in three ways: (1) the T1 peak at 54° at 3 T shifts to 40° at 7 T; (2) the magnitude of the T1 peak is larger at 3 T compared with that at 7 T; and (3) T1 is longer in fibres running parallel to B0 than in those running perpendicular to B0 at 7 T, but not at 3 T.

There are several established MRI methods for measuring T1 values in the brain based either on inversion recovery or saturation recovery. It is apparent that each of these methods produce highly varying GM and WM T1 values, even from the same subjects scanned with the same MRI scanner.^2^ Variation in T1 values is due to several factors, such as flip angle issues, differing sensitivity to B1 inhomogeneity, T2(*) and MT contributions to acquired images. The use of a different kind of T1 sequence may be the reason why Schyboll et al.^22^ reported an angular dependency pattern for T1 at 3 T that is at variance with our recent paper^23^ and the current study. Here, we have used an MP2RAGE sequence with the same inversion and read pulse types at both fields for consistency in T1 quantification. Adiabatic inversion pulses used at both fields will equally invert aqueous proton pools, thereby it is reasonable to assume that initial magnetisations of both myelin-associated and intracellular/extracellular water pools are fully inverted at the very beginning of the inversion recovery (i.e., −1) and that broadband adiabatic inversion will result in a monoexponential inversion recovery curve of aqueous protons.^6^ The read pulses were hard pulses resulting in saturation of semisolid pools at both fields (i.e., MT will contribute to the acquired MP2RAGE signal). The TI range used at both fields will probe long T1 aqueous protons of intracellular/extracellular water almost exclusively. MP2RAGE is an inversion recovery-based MRI sequence that has become a commonplace method to produce T1 maps in the brain at 3 and 7 T. In MP2RAGE, T2*, PD and B1^-^ field biases are removed by combining the T1-weighted and PD-weighted images acquired within the same cycle of inversion recovery. B1^+^ bias is dealt with by separately acquiring B1^+^ maps for correction, as done in this study, or by parameter-specific lookup tables or by incorporating a third RAGE, the so-called MP3RAGE approach.^38^ MP3RAGE was shown to improve T1 quantification in areas where B1^+^ variation at 7 T is an issue. The literature on T1 values estimated by MP2RAGE for GM and WM at 3 and 7 T agree remarkably well with those measured by conventional inversion recovery (IR) MRI.^39^ The T1 values we report for WM with FA of more than 0.45 are in good agreement with those obtained by IR MRI both at 3^1,39^ and 7 T.^40^ While we report that WM T1s at 7 T are somewhat shorter than those reported by Margues et al.,^39^ the WM segmentation used in the latter work was according to T1-weighted signal intensities, whereas we use thresholding according to dMRI microstructural indices. It should be noted that TRs used at 3 T were only ~2.2-times longer than WM T1, whereas at 7 T, TR was more than six times longer than the measured T1. Despite likely T1 saturation of intracellular/extracellular water protons under 3-T data acquisition, the observed WM T1s agree with the literature values.^1,39^ We are confident that the MP2RAGE protocol used here provided unbiased WM T1 data for the purpose of examining the interrelationship between T1 and tissue microstructure.

FA is a scalar proxy for the degree of anisotropy of water diffusion and WM organisation obtained in vivo. It is known to provide only limited information about the microstructural anisotropy imposed by oriented diffusion hindrances, as a multitude of microstructural configurations can lead to identical FA values. In this instance it is worth noting that we have derived FA data using different b-values at 3 and 7 T; however, both b-value ranges used are commonly considered to be optimal, providing comparable quantitative DTI indices.^29^ Hence, dMRI approaches considering fibre orientation dispersion and volume fractions of one or multiple fibre populations might provide a more specific picture of microstructure. Here, referencing WM microstructure by either ODI or F1 assigns WM so that at 3 T, T1 peaks as a function of *θ*_*FB*_ at 54° are approximately 1.5-fold greater than in WM with comparable FA. However, it should be noted that the mean WM T1s across the *θ*_*FB*_ range are indistinguishable by these three microstructural indices. In addition, the size of the T1 peak at the intermediate *θ*_*FB*_ region is greater at 3 than at 7 T. These observations lend support for the conclusion that T1 anisotropy results from physical factors that are either inherent to or generated by myelinated WM axonal components, a finding that is of significance when physical underpinnings are considered.

A large variation in T1 values in the CC is intriguing where WM microstructure mapping by MRI is concerned. In the midline areas of the CC, where fibres run close to 90° to B0, T1 is much longer in areas where the majority of axons are larger than 1 μm and axon density is low, compared with areas where the dominant axonal diameter is smaller than 1 μm and axonal density is high. T1s in genu and splenium ROIs are somewhat lower than those observed in WM globally with comparable FA and ODI at both fields, yet the difference between 3 and 7 T (~100 ms longer T1 at 7 than at 3 T) is similarly seen in global WM. By contrast, in somato-motor ROIs with large diameter fibres, T1s at both fields are in the same range of 910–950 ms. Furthermore, the T1 difference between somato-motor areas and the genu amounts to ~120–150 ms at 3 T^16^ (Figure 8), but to ~70–90 ms at 7 T. These differences cannot be accounted for by the microstructural features probed either by FA or ODI, or orientation-bias with B0 in these areas of the CC, but rather is likely due to high water content and large extra-axonal space in the somato-motor area. A recent study proposed T1 relaxation time as a way of solving crossing fibre configurations for dMRI tractography by collecting T1 maps using both IR MRI and IR-prepared dMRI.^40^ The assumption behind this approach is that T1 varies between tracts due to myelination differences and that T1 from IR-prepared dMRI is multiexponential.^40^ The approach by de Santis et al. also assumes that fibre bundles are homogenous in regard to their other compositions, an assumption that evidently is not the case in the CC.^16^ De Santis et al.^40^ reported that T1 at 7 T in the CST was approximately 140 ms longer than in the CC. A significant proportion of fibres in the CST run close to parallel with B0, whereas fibres in the CC are close to perpendicular to B0. However, our MP2RAGE 7-T T1 data show a much more moderate T1 difference between 0° (where the contribution by the CST is large) and 90° (where the contribution by the CC is large) tracts. Instead, a larger T1 variation within the CC prevails, indicative of variation in axon diameter and density. While myelination and T1 are inversely correlated, we believe that neither myelination differences nor axonal diameter variation play a direct role in the global T1 anisotropy within WM. T1 values from track-based spatial statistics support this conclusion, showing rather uniform relaxation times in individual WM tracts.^23^ Electron microscopy analyses of WM show that the bulk of axonal diameters fall within the range of 0.3–0.6 μm in WM and ‘giant axons’ are found only in some tracts, including the CC and superior longitudinal fasciculus.^41^ In the light of our MRI and electron microscopy data it is reasonable to hypothesise that microstructural features of WM render the magnetic environment such that longitudinal relaxation is affected in an orientation-dependent manner to B0.

While our main objective was to study WM microstructure in relation to angularly dependent T1 signal at 3 and 7 T, our data deserve to be discussed in relation to potential physical underpinnings of T1 anisotropy. In this regard, one can make use of a reference to established angular patterns of relaxation anisotropies for their physical underpinnings. We observe two angular patterns in T1 anisotropy: (1) the 54°/40° peak and (2) B0-dependent gradient between 0° and 90° fibre orientations. It may well be that these two share a common physical mechanism, but equally, they may be attributable to different physical mechanisms. The former feature may be indicative of dipolar effects on protons in a motionally restricted environment resembling ‘the magic angle effect’ on T2 in tendons, cartilage and peripheral nerves.^42,43^ A factor to be considered in regard to the 40°/54° T1 peak is that MT MRI at 3 T has revealed that the ultrashort T2 component in WM with FA of more than 0.7 shows orientation dependency peaking at around 35°–40°. The prolongation of ultrashort T2 has been proposed to result from an orientation-dependent RF absorption imposed by the super-Lorentzian line shape of WM axonal membranes.^33^ Finally, a tissue type that should be considered in interpretation of the underpinnings behind the 40°/54° T1 peak includes small blood vessels that are seen parallel to axon fibres.^44^ Blood oxygenation may have effects on magnetisation in an orientation-dependent manner in this angular range.

For the latter feature, it is interesting that the angular pattern of T1 observed at 7 T resembles that of T2(*).^19,20^ Physical models for the angular dependency of the transverse relaxation in WM have been originally constructed using hollow cylinders for a simplified axon model. In hollow cylinders filled with diamagnetic material, including myelin lipids with anisotropic magnetic susceptibility, that differs chemically from that of their surroundings, diamagnetic susceptibility gradients are generated when placed in an external magnetic field.^19,45^ The size of susceptibility gradients in such a system is determined by several factors, including susceptibility differences across the fibre wall, the orientation of fibres with field and the strength of the external magnetic field.^21^ Thus the local field variations in WM depend on the orientation of fibres with B0. In WM, the longitudinal component of local fields around axonal fibres has been shown to be the source of orientation-dependent static dephasing on T2*,^18,19^ as well as the physical underpinning of the so-called coherence lifetime anisotropy that influences T2.^20,21^ Susceptibility gradients around axons are minimal when fibres are parallel to B0 and maximal at perpendicular orientations, and hence, transverse relaxation is most efficient around fibres running perpendicular to B0. T2 of both myelin-associated and intracellular/extracellular water have been shown to have the same angular dependencies so that T2 is shortest in fibres running perpendicular to B0.^46^ The angular patterns of T2 in both water pools^46^ agree well with that predicted by the hollow cylinder model.^21^

While the longitudinal component of local fields influences transverse relaxation, transverse components of local fields contribute to both longitudinal and transverse relaxation. In the presence of transverse local field components, spins diffusing around fibres will experience field variations around Larmor frequency. This may influence 1/T1 via increased transitions between spin energy eigenstates.^47^ Thus the susceptibility anisotropy and orientation-dependent diffusion of water may also influence the T1 of aqueous water protons. In fact, the T1 data by Schyboll et al. at 3 T,^22^ together with our finding of longer T1 at 7 T in parallel to field fibres rather than those in perpendicular orientations, fit with this effect. As mentioned earlier, each T1 sequence produces characteristic in vivo T1,^2^ and it may well be that each T1 protocol is influenced differently by physical mechanisms as well. If so, it is expected that angular patterns of T1 in WM as measured by VFA and MP2RAGE are not alike, for instance, reflecting their inherent sensitivities to local field variations. Schyboll et al.^48^ used molecular dynamics simulations to estimate the effects of local field variation to myelin-associated water as a potential source for the observed T1 relaxation anisotropy. Their simulations indicate an effect by local field variation on the orientation-dependent T1 in myelin-associated water; however, this effect may be too small in vivo WM to account for the observed angular pattern of T1 at 3 T. It should be noted that the modelling by Schyboll et al.^48^ focused on myelin-associated water, which may have a T1 in the low hundreds of milliseconds, rather than on ‘bulk water’, the dominant source of WM MRI signal. Nevertheless, the angular plots Schyboll et al.^22^ reported for VFA T1 at 3 T are tantalisingly similar to those we observed for MP2RAGE T1 at 7 T. Instead, Schyboll et al.^49^ proposed a dipolar model for interactions between myelin-associated water and solid myelin protons that may account for T1 shortening in fibres close to or at 90° with respect to B0.

To conclude, the current T1 data from 3 and 7 T show T1 anisotropy in WM with a high degree of structural anisotropy and ordered fibre structure. This anisotropy is characterised by two features in angular plots of T1: first, a peak with a long T1 centred around ~40° and ~54° of *θ*_*FB*_ at 7 and 3 T, respectively, and second, a longer T1 in parallel to B0 fibres than perpendicular to B0 fibres at 7 T. Regarding axonal diameter and density, our data from the CC show longer T1s in areas of the CC where the axon diameter exceeds 1 μm and axon density is low than in areas with a dominant proportion of small axons tightly packed. The effect of axon fibre diameter and density appear to be B0-dependent so that the difference in T1 between WM with large axons and that with small fibres is less by ~40% at 7 than at 3 T. Our data strongly link T1 relaxation in WM to microstructure, indicating that myelinated fibres play a dual role in T1 contrast, and show that T1 may provide a more comprehensive description of brain tissue, complementary to microstructural characteristics from dMRI. Advanced hardware, such as a tiltable headcoil,^50^ are expected to be useful in future studies on the interrelationship between WM microstructure and MRI relaxations.

## Supplementary Material

SUPINFO

## Figures and Tables

**TABLE 1:** Diffusion MRI acquisition parameters at 3 and 7 T

Parameter	3 T	7 T
Voxel size (mm)	1.5 × 1.5 × 1.5	1.05 × 1.05 × 1.05
Slices	92	128
TR (ms)	3230	7000
TE (ms)	89.20	71.20
Multiband acceleration	4	2
GRAPPA	-	3
Phase-encoding direction	A>>P, P>>A	A>>P, P>>A
Gradient directions	197 (AP), 197 (PA)	143 (AP), 143 (PA)
*b-*values (s/mm^2^)	1500, 3000	1000, 2000
Number of *b* = 0 s/mm^2^ volumes	13 (AP), 17 (PA)	11 (AP), 13 (PA)
Total acquisition (min:s)	22:38	39:48

Abbreviations: AP, anterior-posterior; GRAPPA, GeneRalized Autocalibrating Partial Parallel Acquisition; PA, posterior-anterior.

**TABLE 2:** Summary of numerical data from T1 versus the fibre-to-field-angle plots for each microstructural index at 3 and 7 T

					T1	T1	ΔT1	ΔT1	ΔT1	ΔT1
Parameter	3 T	7 T	FA 3 T	FA 7 T	(ms) 3 T	(ms) 7 T	(54°)3T	(40°)7T	(0°–90°)3T	(0°–90°)7T
FA	0.725	0.725	-	-	817.6 ± 15.1	905.0 ± 26.0	38.7 ± 11.1	22.4 ± 4.2	−4.6 ± 21.1	47.2 ± 20.2*
F1	0.519	0.550	0.755 ± 0.08	0.685 ± 0.007	815.0 ± 22.8	912.7 ± 15.6	56.4 ± 19.0*	26.8 ± 4.0	9.9 ± 27.9	49.1 ± 35.1
F2	0.312	0.331	0.779 ± 0.031	0.672 ± 0.026	804.7 ± 23.0	914.4 ± 14.7	48.0 ± 18.0*	12.9 ± 6.2	12.9 ± 20.0	35.4 ± 29.4
F3	0.238	0.206	0.602 ± 0.009	0.524 ± 0.015	817.9 ± 9.7	924.9 ± 4.1	10.4 ± 6.1	4.5 ± 3.2	−11.4 ± 24.4	9.7 ± 13.4
ODI	0.05	0.05	0.772 ± 0.015	0.788 ± 0.015	817.8 ± 24.4	907.1 ± 26.5	59.4 ± 17.2*	37.0 ± 7.5	17.2 ± 19.7	75.6 ± 25.2*

*Note*: The values in the 3 and 7 T columns are the medians for the given microstructural index shown in the row. ΔT1 (54°) indicates the difference in T1 between 54° and the baseline at 3 T; ΔT1 (40°) indicates the difference in T1 between 40° and the baseline at 7 T; ΔT1 (0–90°) indicates the difference in T1 between the mean T1 at *θ*_FB_ of (0° + 4.5°)/2 and at *θ*_FB_ of (85° + 90°)/2 at both fields. The baseline in T1 versus *θ*_*FB*_ for each microstructural index was taken to be the straight line joining T1 points between the means of T1s from 0° to 20° and 80° to 90°. Values are mean ± SD. The number of datasets was 11 at 3 T and six at 7 T. T1 is given in ms. Student’s *t*-test is used for ΔT1 (54°) and ΔT1 (0°–90°) between 3 and 7 T. The Bonferroni-corrected *p* value is set at 0.01. An asterix shows a significant difference.

Abbreviations: FA, fractional anisotropy; F1, one-fibre orientation volume fraction; F2, two-fibre orientation volume fraction; F3, three-fibre orientation volume fraction; ODI, orientation dispersion index.

## Abbreviations used:

CC: corpus callosum
CST: cortico-spinal tract
GM: grey matter
F1: one-fibre orientation volume fraction
F2: two-fibre orientation volume fraction
F3: three-fibre orientation volume fraction
HCP: Human Connectome Project
NODDI: Neurite Orientation and Density Imaging
ODI: orientations dispersion index
θ_FB_: fibre-to-field angle
SNR: signal-to-noise-ratio
VFA: variable flip angle
WM: white matter
