# Supplementary material for: White matter microstructure and longitudinal relaxation time anisotropy in human brain at 3 and 7 T
Source: NMR Biomed. 2022 Sep 12;36(1):e4815. doi: 10.1002/nbm.4815 (PMC9742158; doi:10.1002/nbm.4815)
Supplement: Supplementary file 1 — Figure S1. 1D plots for T1 as a function of fibre‐to‐field angle in WM with median FA = 0.725. MRI data are from those six volunteers scanned at both 3T and 7T. In (A) T1 and DTI data were from 7T. In (B) the 7T T1 maps were down‐sampled to 3T resolution, registered on 3T FA maps and these T1 maps plotted for fibre‐to‐field angles using 3T DTI data; in (C) T1 and DTI 3T data from the volunteers were plotted for T1 as function of the fibre‐to‐field angle. Red arrows point to the peak T1 values. Figure S2. Signal‐to‐noise ratios in diffusion MR images. SNR was estimated from a ROI encompassing a section along the entire midsagittal antero‐posterior length of the corpus callosum (CC) using the procedure described in 1. SNR values (mean ± SD) in panel A are for 3T and in panel B for 7T images. The bars in both panels are labelled from left to right as follows: b0 (b = 0 s/mm2), X (X gradient direction), Y (Y gradient direction) and Z (Z gradient direction). In panel A the blue bars are for b = 1,500 s/mm2, the brown bars for 3,000 s/mm2 and in panel B the green bars for b = 1,000 s/mm2 and the red bars for b = 2,000 s/mm2 images. [file NBM-36-0-s001.docx]

**WHITE MATTER MICROSTRUCTURE AND LONGITUDINAL RELAXATION TIME ANISOTORPY IN HUMAN BRAIN AT 3T AND 7T**

Risto A. Kauppinen^1^, Jeromy Thotland^2^, Pramod Pisharady^2^, Christophe Lenglet^2^ and Michael Garwood^2^

^1^Department of Electric and Electronic Engineering, University of Bristol, Bristol, UK and ^2^Center for Magnetic Resonance Research, University of Minnesota, Minneapolis, MN, USA


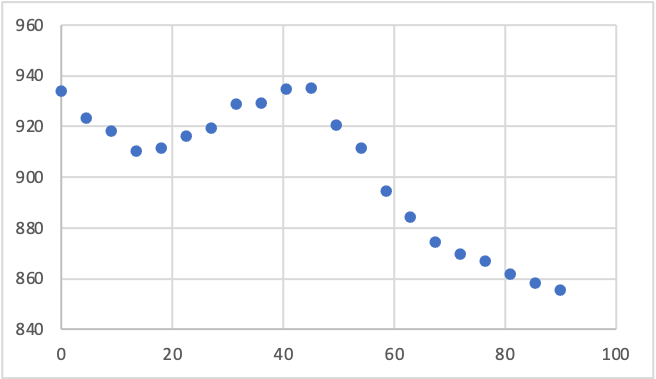

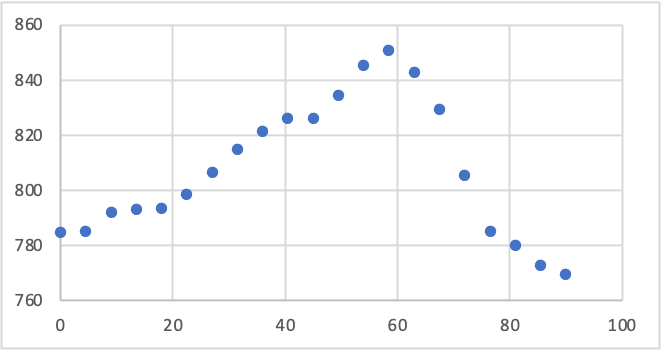

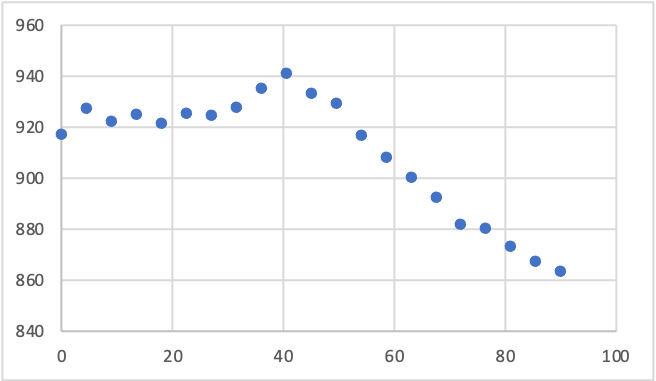

$$\boldsymbol{\theta}$$

**A**

**B**

**C**

**T_1_**

**T_1_**

**T_1_**

**Figure S1.** 1D plots for T1 as a function of fibre-to-field angle in WM with median FA = 0.725. MRI data are from those six volunteers scanned at both 3T and 7T. In (A) T1 and DTI data were from 7T. In (B) the 7T T1 maps were down-sampled to 3T resolution, registered on 3T FA maps and these T1 maps plotted for fibre-to-field angles using 3T DTI data; in (C) T1 and DTI 3T data from the volunteers were plotted for T1 as function of the fibre-to-field angle. Red arrows point to the peak T1 values.


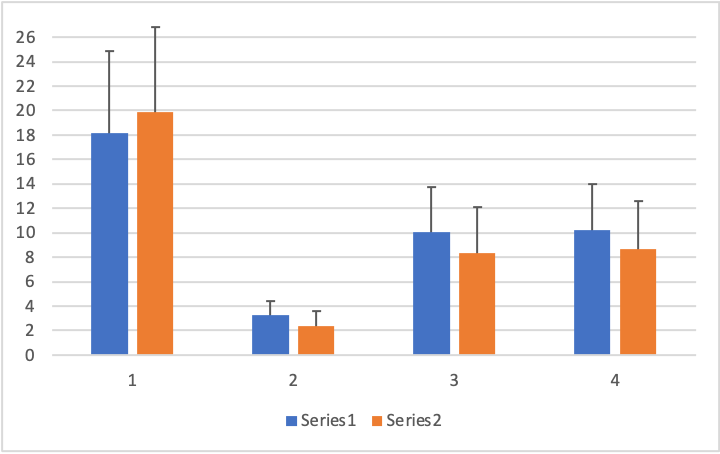

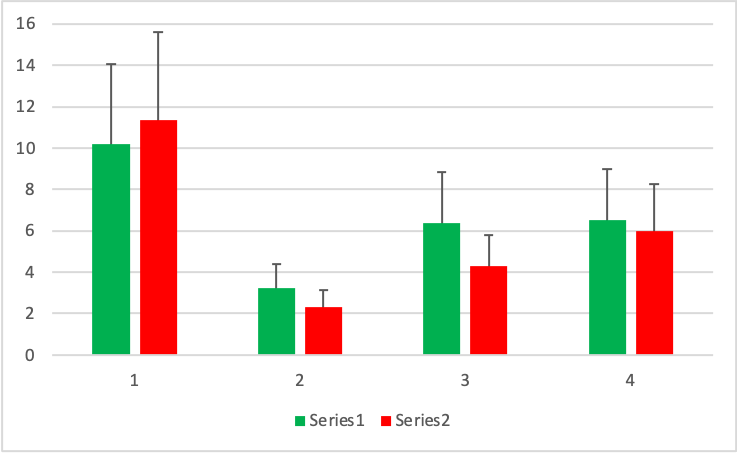


**SNR**

**SNR**

**b0 X Y Z**

**A**

**B**

**Figure 2S. Signal-to-noise ratios in diffusion MR images.**

SNR was estimated from a ROI encompassing a section along the entire midsagittal antero-posterior length of the corpus callosum (CC) using the procedure described in ^1^. SNR values (mean ± SD) in panel A are for 3T and in panel B for 7T images. The bars in both panels are labelled from left to right as follows: b0 (b = 0 s/mm^2^), X (X gradient direction), Y (Y gradient direction) and Z (Z gradient direction). In panel A the blue bars are for b = 1,500 s/mm^2^, the brown bars for 3,000 s/mm^2^ and in panel B the green bars for b = 1,000 s/mm^2^ and the red bars for b = 2,000 s/mm^2^ images.

**REFERENCES**

1. Descoteaux M, Deriche R, Le Bihan D, Mangin JF, Poupon C. Multiple q-shell diffusion propagator imaging. *Med Image Anal*. 2011;15:603-21.
